# Supplementary material for: MetaRibo-Seq measures translation in microbiomes
Source: Nat Commun. 2020 Jun 29;11:3268. doi: 10.1038/s41467-020-17081-z (PMC7324362; doi:10.1038/s41467-020-17081-z)
Supplement: Supplementary file 10 — Supplementary Data 7 [file 41467_2020_17081_MOESM10_ESM.zip › File2/Confidence_High_Taxonomy/1417_out.krona.html]

Javascript must be enabled to view this page.

members
magnitude
magnitudeUnassigned
count
unassigned
taxon
rank

1417\_out

110

2
110
superkingdom

phylum
976
110

200643
110
class

171549
110
order

110
815
family

1
816
genus

SRS075773\_contig\_number\_30284
110

species
1

SRS014979\_contig\_number\_34189
28116

85

SRS012273\_contig\_number\_47288SRS012849\_contig\_number\_contig-100\_1459.190981SRS013476\_contig\_number\_contig-100\_2228.207207SRS013687\_contig\_number\_4200SRS013965\_contig\_number\_38418SRS014287\_contig\_number\_contig-100\_1578.113591SRS014412\_contig\_number\_contig-100\_6835.106608SRS014923\_contig\_number\_33435SRS014948\_contig\_number\_contig-100\_1657.120158SRS015264\_contig\_number\_21338SRS016056\_contig\_number\_7843SRS016203\_contig\_number\_10804SRS018049\_contig\_number\_761SRS018313\_contig\_number\_1933SRS018541\_contig\_number\_9701SRS018936\_contig\_number\_contig-100\_1602.80301SRS019030\_contig\_number\_contig-100\_1426.110365SRS019267\_contig\_number\_5654SRS019397\_contig\_number\_735SRS019601\_contig\_number\_153SRS024492\_contig\_number\_12303SRS042628\_contig\_number\_14873SRS042966\_contig\_number\_contig-100\_1553.120238SRS043701\_contig\_number\_15828SRS043841\_contig\_number\_11047SRS046068\_contig\_number\_contig-100\_1765.36511SRS047044\_contig\_number\_15491SRS049712\_contig\_number\_22057SRS049773\_contig\_number\_29349SRS050299\_contig\_number\_370SRS050752\_contig\_number\_7658SRS050941\_contig\_number\_21083SRS050998\_contig\_number\_17317SRS051882\_contig\_number\_16382SRS056259\_contig\_number\_21428SRS057480\_contig\_number\_contig-100\_4551.72653SRS058770\_contig\_number\_2007SRS062654\_contig\_number\_549SRS063518\_contig\_number\_contig-100\_1230.204497SRS065504\_contig\_number\_21515SRS074964\_contig\_number\_6241SRS076756\_contig\_number\_contig-100\_6326.121266SRS077024\_contig\_number\_contig-100\_1481.87031SRS077294\_contig\_number\_7402SRS077502\_contig\_number\_18628SRS077849\_contig\_number\_813SRS078176\_contig\_number\_2897SRS078242\_contig\_number\_5272SRS098571\_contig\_number\_181SRS098644\_contig\_number\_395SRS100907\_contig\_number\_8544SRS101268\_contig\_number\_721SRS1041031\_contig\_number\_14158SRS1041039\_contig\_number\_contig-100\_1827.147052SRS1041090\_contig\_number\_contig-100\_1355.51422SRS1041091\_contig\_number\_10580SRS1041137\_contig\_number\_4487SRS1041141\_contig\_number\_14933SRS1041145\_contig\_number\_17151SRS104400\_contig\_number\_39485SRS1055022\_contig\_number\_24219SRS1055049\_contig\_number\_4198SRS142599\_contig\_number\_16766SRS142781\_contig\_number\_2298SRS143342\_contig\_number\_208SRS143523\_contig\_number\_23336SRS143876\_contig\_number\_5534SRS144603\_contig\_number\_17014SRS147039\_contig\_number\_4382SRS147557\_contig\_number\_28993SRS147557\_contig\_number\_contig-100\_279.177253SRS147614\_contig\_number\_contig-100\_1813.54454SRS147977\_contig\_number\_8480SRS148196\_contig\_number\_8737SRS148253\_contig\_number\_3317SRS148319\_contig\_number\_contig-100\_1540.94177SRS148424\_contig\_number\_contig-100\_2020.170611SRS148817\_contig\_number\_contig-100\_4453.4454SRS149244\_contig\_number\_81SRS149784\_contig\_number\_contig-100\_1368.153222SRS149879\_contig\_number\_25577SRS150029\_contig\_number\_contig-100\_2078.104208SRS892882\_contig\_number\_contig-100\_399.96396SRS893285\_contig\_number\_235SRS893383\_contig\_number\_34222
47678
species


SRS048164\_contig\_number\_12214
28111
1
species

13
626931

SRS016335\_contig\_number\_contig-100\_9141.9141SRS017191\_contig\_number\_contig-100\_2245.112871SRS019178\_contig\_number\_contig-100\_1452.36172SRS021484\_contig\_number\_2701SRS023914\_contig\_number\_14716SRS043001\_contig\_number\_1834SRS048060\_contig\_number\_12943SRS062427\_contig\_number\_8841SRS065397\_contig\_number\_contig-100\_6431.6432SRS077024\_contig\_number\_148SRS104975\_contig\_number\_contig-100\_1452.122221SRS143598\_contig\_number\_1572SRS147346\_contig\_number\_43849
species

species
2

SRS013965\_contig\_number\_40392SRS052697\_contig\_number\_25847
818

1946091

SRS1041133\_contig\_number\_17098
1
species

species
4

SRS044535\_contig\_number\_contig-100\_6669.6670SRS1054716\_contig\_number\_9643SRS893292\_contig\_number\_2963SRS893300\_contig\_number\_9521
338188

821

SRS054905\_contig\_number\_20799
1
species

species

SRS015663\_contig\_number\_482
310298
1
